# Different association between triglyceride-glucose index and mild cognitive impairment in type 2 diabetes mellitus patients with and without diabetic kidney disease

**DOI:** 10.3389/fnut.2025.1681164

**Published:** 2025-11-12

**Authors:** Songtao Feng, Zhen Zhang, Qingyue Gao, Ling Huang, Ya Zhang, Mengting He, Bing Song, Wenwen Zhu, Li Ding

**Affiliations:** 1 Department of Nephrology, Jiangsu University Affiliated People’s Hospital, Zhenjiang, China; 2 Department of Endocrinology and Metabolism, The First Affiliated Hospital of USTC, Division of Life Sciences and Medicine, University of Science and Technology of China, Hefei, China; 3 Department of Endocrinology and Metabolism, Anhui Provincial Hospital Affiliated to Bengbu Medical University, Hefei, China; 4 Department of Endocrinology, The First Affiliated Hospital of Jinzhou Medical University, Jinzhou, China; 5 Department of Endocrinology, Huzhou Central Hospital, Affiliated Central Hospital of Huzhou University, Fifth School of Clinical Medicine of Zhejiang Chinese Medical University, Huzhou, China; 6 Department of Endocrinology, Taikang Xianlin Drum Tower Hospital, Affiliated Hospital of Medical School, Nanjing University, Nanjing, China

**Keywords:** triglyceride-glucose index, diabetic kidney disease, mild cognitive impairment, type 2 diabetes mellitus, diabetic complication

## Abstract

**Aim:**

The triglyceride-glucose (TyG) index, a surrogate marker reflecting metabolic status related to both glucose and lipid homeostasis, has been implicated in the development of diabetes-related complications, including diabetic kidney disease (DKD). Metabolic disturbances in carbohydrate and lipid pathways have also been linked to impairments in cognitive performance. This study aims to explore the association between TyG levels and the presence of mild cognitive impairment (MCI) among individuals with type 2 diabetes mellitus (T2DM), stratified by DKD status.

**Methods:**

A total of 243 patients with T2DM were divided into two subgroups based on cognitive status: those with MCI and those without. Clinical parameters were assessed and compared between the two cohorts. The association between TyG index and cognitive function was analyzed. Furthermore, potential predictors of MCI were explored separately in T2DM patients with and without DKD.

**Results:**

In individuals with T2DM, those exhibiting MCI (*n* = 95) showed significantly higher TyG index values in comparison to counterparts with normal cognition (*n* = 148). Elevated TyG index was linked to reduced performance on the Montreal Cognitive Assessment, a measure of global cognition, as well as diminished scores on both the Verbal Fluency Test and the delayed recall component of the Auditory Verbal Learning Test, which assess executive function and immediate memory, respectively, in patients without DKD. These findings suggest that heightened TyG index may serve as an independent risk indicator for cognitive decline in T2DM individuals without DKD. However, among those with coexisting DKD, no statistically meaningful association between TyG index and cognitive outcomes was observed.

**Conclusion:**

Elevated TyG index levels have been linked to an increased likelihood of MCI, particularly affecting executive function and immediate recall, among individuals with T2DM but without DKD.

## 1 Introduction

Type 2 diabetes mellitus (T2DM) is a chronic metabolic disorder characterized by reduced insulin efficacy and pancreatic *β*-cell impairment (1). Beyond its hallmark disruption of glucose regulation, it is often accompanied by significant alterations in lipid profiles (2, 3). The global burden of T2DM continues to rise. According to the 2021 report from the International Diabetes Federation, over 530 million people worldwide are living with diabetes, with T2DM accounting for over 90% of cases (4). China is among the countries most affected, with national epidemiological data indicating a diabetes prevalence of approximately 12.8% among adults aged 18 and above. This growing trend poses a considerable challenge to public health systems (5). As the diabetic population expands, complications such as mild cognitive impairment (MCI) have garnered increasing attention from researchers (6). The development of MCI is multifactorial, involving disruptions in metabolic homeostasis—particularly insulin resistance-related abnormalities in glucose and lipid pathways—alongside mechanisms such as neuroinflammation, oxidative stress, and neuronal loss (7). Among these contributors, dysregulation of glucose and lipid metabolism is believed to play a pivotal initiating role.

The triglyceride-glucose (TyG) index, an integrated marker reflecting both glucose and lipid metabolic dysfunction, has emerged in recent years as a valuable tool in the assessment of chronic complications associated with diabetes including diabetic retinopathy, diabetic nephropathy, diabetic cardiovascular disease (8–10). Studies have shown that the TyG index not only reflects disturbances in glucose and lipid metabolism but is also closely associated with insulin resistance (11), which serves as a common risk factor for both diabetes and cognitive impairment (12). Previously, we have described a link between dysregulation of glucose and lipid metabolism and cognitive impairment in individuals with diabetes. Interestingly, recent investigations have extended this association to non-diabetic populations, where the glucose-lipid metabolic index has also demonstrated a significant correlation with cognitive decline (13). Moreover, clinical studies have revealed that not only the TyG index (14, 15), but also its derivatives—such as the TyG index adjusted by waist-to-height ratio (16) and the TyG index combined with body mass index (BMI) (17) —are associated with impaired cognitive function. These observations have been further supported by database analyses (18) and meta- analyses (19, 20). Although the association between the TyG index and specific cognitive domains has not been thoroughly examined, prior studies have identified a correlation between the TyG index and MCI in individuals with T2DM (21), suggesting its potential as a biomarker for MCI detection (22).

A study has suggested a potential association between chronic kidney disease and the TyG index in individuals with diabetes (23). Indeed, emerging evidence indicates that the TyG index may not only be correlated with diabetic kidney disease (DKD) but could also serve as a candidate biomarker for its diagnosis (24). Studies have shown that cognitive dysfunction in diabetes is closely associated with microvascular complications (25–27). As previously mentioned, diabetic microvascular diseases—including diabetic nephropathy and diabetic retinopathy—are strongly linked to TyG index levels. Indeed, our recent findings also suggest that patients with T2DM and coexisting diabetic nephropathy may be more susceptible to MCI (28).

While prior investigations have examined the association between the TyG index and cognitive impairment in individuals with T2DM, few have addressed its relationship with specific cognitive domains beyond global cognitive decline. Moreover, considering the well-established link between diabetic microvascular complications—particularly DKD—and cognitive dysfunction, as well as the complex and significant connection between DKD and the TyG index, our study aimed to provide a more nuanced analysis. Specifically, we evaluated the association between the TyG index and various dimensions of MCI in patients with T2DM. Furthermore, we conducted stratified analyses based on the presence or absence of DKD to determine whether the TyG index demonstrated differential associations with cognitive performance in these subgroups.

## 2 Methods

### 2.1 Study design and ethical approval

A total of 243 patients diagnosed with T2DM were recruited from the Department of Endocrinology at the First Affiliated Hospital of USTC. Of these, 95 individuals exhibiting MCI were assigned to the MCI group, while the remaining 148 participants, who demonstrated normal cognitive performance, comprised the control group. Diabetes was identified based on the diagnostic criteria outlined in the 1999 World Health Organization guidelines (29). The diagnosis of DKD was documented in the electronic medical records by clinicians in accordance with the standards set by the American Diabetes Association (30). MCI in the study participants was identified in accordance with the diagnostic criteria established by the MCI Working Group under the European Consortium for Alzheimer’s Disease (31). All data usage was covered under a broad informed consent (Since this retrospective study did not involve any additional blood sample collection, according to the ethics committee’s regulations, it was not necessary to inform patients of the specific research procedure) signed by the patients. The study protocol was reviewed and approved by the Ethics Committee of the First Affiliated Hospital of USTC (Approval No.: 2023-RE-292).

### 2.2 Inclusion and exclusion criteria

This study enrolled individuals diagnosed with T2DM for a duration exceeding 3 years. Participants were screened based on specific exclusion criteria relevant to DKD, including: (a) diabetes types other than T2DM; (b) occurrence of acute metabolic complications, such as diabetic ketoacidosis, hyperosmolar hyperglycemic syndrome, or lactic acidosis; (c) prior episodes of severe hypoglycemia; (d) major cardiovascular or cerebrovascular events; (e) additional vascular pathologies; (f) conditions like venous thromboembolism or lymphangitis; (g) any history of limb amputation; (h) abnormalities in thyroid function; (i) tobacco use; (j) alcohol intake; and (k) administration of drugs that may impair renal function.

### 2.3 Clinical data

Demographic and clinical characteristics, including age, sex, diabetes mellitus duration (DMM), and hypertension, were systematically extracted from patients’ medical records. Upon hospital admission, anthropometric assessments were performed, recording height and weight to calculate BMI using the standard formula: body mass (kg) divided by height squared (m^2^). On the second day post-admission, venous blood samples were drawn for biochemical profiling. Glucose metabolism was evaluated by measuring glycated hemoglobin (HbA1c), and fasting plasma glucose (FPG). Lipid parameters assessed included triglycerides (TG), total cholesterol (TC), low-density lipoprotein cholesterol (LDL-C), and high-density lipoprotein cholesterol (HDL-C). Renal function was assessed through serum creatinine (Cr) and uric acid (UA) concentrations. All laboratory analyses were performed in the Central Laboratory of the First Affiliated Hospital of the University of Science and Technology of China (USTC). The estimated glomerular filtration rate (eGFR) was calculated based on serum Cr values. TyG index was computed using the equation: Ln [fasting TG (mg/dL) × fasting glucose (mg/dL)/2]. In addition, urine samples were analyzed for microalbumin and creatinine to determine the urinary albumin-to-creatinine ratio (UACR). All relevant clinical and laboratory data were retrieved from institutional electronic medical records.

### 2.4 Neurocognitive performance

Neurocognitive abilities were assessed through a comprehensive series of validated instruments aligned with established research guidelines (32, 33). Overall cognitive capacity was determined using the Montreal Cognitive Assessment (MoCA), with an additional score adjustment granted to participants with fewer than 12 years of formal education, following standard correction protocols. Information processing speed function was measured via the Trail Making Test Part A (TMTA), whereas executive function was evaluated using a combination of the Digit Span Test (DST), Verbal Fluency Test (VFT), and Trail Making Test Part B (TMTB). Memory performance, including both immediate and delayed recall, was examined using the Auditory Verbal Learning Test (AVLT), incorporating its Immediate Recall (AVLT-IR) and Delayed Recall (AVLT-DR) components. Contextual or story-based memory was further explored using the Logical Memory Test (LMT) (34–38).

### 2.5 Statistical methods

All statistical analyses were carried out using SPSS software (version 22.0; IBM, United States). Variables conforming to a normal distribution, such as TC and LDL-C, were expressed as mean values with standard deviations and compared across groups using independent sample t-tests. For non-normally distributed variables—including age, DMM, BMI, HbA1c, FPG, TG, HDL-C, TyG, UA, serum Cr, eGFR, and UACR—data were presented as medians with interquartile ranges, and intergroup differences were assessed using the Mann–Whitney U test. Categorical variables, including sex, as well as presence of DKD and hypertension, were summarized as frequencies and proportions, with comparisons made via the chi-square test. To assess associations between variables, both Pearson correlation and partial correlation analyses were conducted, with and without adjustment for potential confounders. In addition, binary logistic regression was applied to explore risk factors for MCI in T2DM patients with and without DKD.

## 3 Results

### 3.1 Analysis of clinical features among T2DM individuals with or without MCI

This study commenced with an evaluation of the clinical characteristics of T2DM patients, stratified by the presence or absence of MCI. Although the investigation was cross-sectional and the two groups were not rigorously matched, no significant differences emerged in baseline parameters such as age, gender, DMM or hypertension. Similarly, BMI, TC, TG, LDL-c, and HDL-c showed no statistical divergence between the 2 groups (all *p* > 0.05). In contrast, individuals in the MCI subgroup displayed higher levels of HbA1c and FPG than those without cognitive deficits (all *p* < 0.05). To further explore metabolic differences, the TyG index was calculated as an integrated indicator of glucose and lipid dysregulation. The primary objective was to assess whether TyG is implicated in the cognitive impairment observed among T2DM patients. Accordingly, TyG values and renal function indicators—including UA, Cr, eGFR, and UACR—were compared between the two groups. The findings revealed that patients with MCI exhibited significantly elevated TyG levels compared to those without MCI (all *p* < 0.05). Furthermore, UACR values were notably increased in the MCI group relative to those without DKD (all *p* < 0.05), while no significant differences were observed in UA, Cr, or eGFR between the groups (refer to Table 1).

**Table 1:** Comparation of clinical parameters and cognitive performance between Non-MCI group and MCI group.

	Non-MCI (*n* = 148)	MCI (*n* = 95)	*P*
Age (year)	62.00 (56.00, 70.75)	61.00 (56.00, 70.00)	0.710 ^a^
Female (n, %)	71, 47.97	39, 41.05	0.290 ^c^
DDM (year)	10.00 (6.00, 18.00)	10.00 (8.00, 17.00)	0.627 ^a^
Hypertension (n, %)	68, 45.95	52, 54.74	0.181 ^a^
BMI (kg/m^2^)	24.39 (20.05, 26.16)	24.44 (22.49, 26.78)	0.959 ^a^
HbA1c (%)	7.65 (6.90, 8.80)	8.40 (7.10, 9.70)	0.024 ^a*^
FPG (mmol/L)	6.83 (5.49, 8.41)	8.18 (6.36, 10.23)	0.012 ^a*^
TG (mmol/L)	1.41 (0.99, 1.99)	1.56 (1.02, 2.34)	0.103 ^a^
TC (mmol/L)	4.46 ± 1.02	4.36 ± 1.11	0.241 ^b^
LDL-c (mmol/L)	2.66 ± 0.79	2.60 ± 0.87	0.297 ^b^
HDL-c (mmol/L)	1.12 (0.94, 1.29)	1.04 (0.88, 1.20)	0.127 ^a^
TyG	9.60 (9.19, 10.08)	9.92 (9.36, 10.37)	0.031 ^a*^
UA (μmol/L)	304.05 (256.88, 353.65)	310.00 (259.00, 359.20)	0.753 ^a^
Cr (μmol/L)	63.00 (54.00, 74.00)	65.00 (53.00, 82.00)	0.440 ^a^
eGFR (mL/min/1.73 m^2^)	94.28 (86.07, 102.44)	94.25 (79.86, 103.91)	0.959 ^a^
UACR (mg/g)	13.21 (6.85, 31.14)	32.43 (11.19, 115.17)	0.003 ^a*^
DKD (n, %)	40, 20.03	51, 53.68	<0.001 ^c*^
MoCA	28.00 (27.00, 29.00)	25.00 (23.00, 25.00)	<0.001 ^a*^
DST	12.00 (11.00, 15.00)	11.00 (8.00, 12.00)	0.002 ^a*^
VFT	17.00 (14.00, 20.00)	12.00 (10.00, 15.00)	<0.001 ^a*^
CDT	3.00 (2.00, 4.00)	2.00 (2.00, 3.00)	<0.001 ^a*^
TMTA	58.50 (49.00, 73.75)	72.00 (58.00, 84.00)	<0.001 ^a*^
TMTB	152.50 (116.00, 188.75)	182.00 (147.00, 214.00)	<0.001 ^a*^
AVLT-IR	18.00 (14.00, 21.00)	15.00 (12.00, 18.00)	0.006 ^a*^
AVLT-DR	8.00 (6.00, 9.75)	6.00 (5.00, 8.00)	<0.001 ^a*^
LMT	9.00 (6.00, 12.00)	7.00 (4.00, 9.00)	0.002 ^a*^

^a^The Mann–Whitney U test was employed for asymmetrically distributed variables; ^b^Student’s *t*-test was employed for normally distributed variables; ^c^The Chi-square test was employed for categorical variables. ^*^p < 0.05. MCI, mild cognitive impairment; DDM, duration of diabetes mellitus; BMI, body mass index; HbA1c, glycosylated hemoglobin; FPG, fasting plasma glucose; TG, triglycerides; TC, total cholesterol; LDL-c, low density lipoprotein cholesterol; HDL-c, high density lipoprotein cholesterol; TyG, triglyceride glucose index; UA, uric acid; Cr, creatinine; eGFR, Estimated glomerular filtration rate; DKD, diabetic kidney disease; MoCA, Montreal cognitive assessment; DST, digit span test; VFT, verbal fluency test; CDT, clock drawing test; TMTA, trail making test-A; TMTB, trail making test-B; AVLT-IR, auditory verbal learning test-immediate recall; AVLT-DR, auditory verbal learning test-delayed recall; LMT, logical memory test.

### 3.2 Comparison of neurocognitive performance in T2DM patients with and without MCI

In order to thoroughly assess cognitive deficits among individuals with MCI, we conducted a comparative analysis of both overall and domain-specific neuropsychological performance in T2DM patients with and without MCI. Global cognitive capacity was evaluated using the MoCA, while a battery of specialized tests—namely DST, VFT, CDT, TMTA, TMTB, AVLT-IR, AVLT-DR, and LMT—was employed to examine specific cognitive domains. Findings revealed that those in the T2DM group with coexisting MCI showed significantly lower MoCA scores (*p* < 0.001), indicative of diminished general cognitive function. In addition, poorer performance on DST, VFT, and TMTB suggested executive dysfunction, while scores on CDT and TMTA pointed to deficits in visuospatial ability and processing speed, respectively (all *p* < 0.05). Reductions in AVLT-IR and AVLT-DR were associated with impairments in short-term and delayed verbal memory, and decreased LMT scores indicated a decline in contextual memory abilities (all *p* < 0.05) (refer to Table 1).

### 3.3 Association between TyG and neurocognitive performance in patients with T2DM

To investigate the association between the TyG index and cognitive performance in individuals with T2DM, Pearson correlation analyses were performed. The findings demonstrated that higher TyG values were significantly associated with lower scores on MoCA, which reflects global cognitive function (*R* = −0.173, *p* = 0.007), and on VFT, indicative of executive function (*R* = −0.196, *p* = 0.002). In contrast, TyG showed a significant negative correlation with AVLT-IR (*R* = −0.171, *p* = 0.011), a measure related to memory function. These relationships remained robust in partial correlation analyses controlling for age, sex, and DDM. Specifically, the correlation between TyG and DST was −0.126 (*p* = 0.050) before adjustment, and slightly strengthened to −0.128 (*p* = 0.048) after adjusting for the aforementioned covariates. (see Table 2).

**Table 2:** Association between TyG and cognitive performance in patients with T2DM.

	Model 1		Model 2	
	R	*P*	R	*P*
MoCA	−0.173	0.007 ^*^	−0.197	0.002 ^*^
DST	−0.126	0.050	−0.128	0.048 ^*^
VFT	−0.196	0.002 ^*^	−0.189	0.003 ^*^
CDT	−0.021	0.744	−0.048	0.456
TMTA	0.010	0.877	0.017	0.798
TMTB	0.098	0.129	0.098	0.131
AVLT-IR	−0.146	0.023 ^*^	−0.155	0.017 ^*^
AVLT-DR	−0.097	0.133	−0.093	0.151
LMT	0.026	0.687	−0.004	0.951

^*^P < 0.05. Model 1 showed the Pearson association between TyG and cognitive performance in patients with T2DM; Model 2 showed the partial association between TyG and cognitive performance in patients with T2DM adjusting for age and gender as well as duration of diabetes mellitus. TyG, triglyceride glucose index; T2DM, type 2 diabetes mellitus; MoCA, Montreal cognitive assessment; DST, digit span test; VFT, verbal fluency test; CDT, clock drawing test; TMTA, trail making test-A; TMTB, trail making test-B; AVLT-IR, auditory verbal learning test-immediate recall; AVLT-DR, auditory verbal learning test-delayed recall; LMT, logical memory test.

### 3.4 Difference of the relationship between TyG and neurocognitive performance in T2DM patients with and without DKD

To explore whether the relationship between the TyG index and cognitive function differs according to DKD status, we performed a stratified subgroup analysis based on the presence or absence of DKD. The results demonstrated marked heterogeneity between individuals with T2DM depending on DKD status. Among those without DKD, elevated TyG levels were significantly associated with poorer performance on several neuropsychological tests, including MoCA, VFT, and AVLT-IR (all *p* < 0.05). Conversely, these associations were not evident in participants with DKD (all *p* > 0.05) (see Supplementary Table 1). Importantly, after controlling for gender, age, and DMM via partial correlation analyses, the inverse correlations between TyG and scores on the MoCA, VFT, and AVLT-IR remained statistically significant in the non-DKD group (all *p* < 0.05) (see Table 3).

**Table 3:** Partial association between TyG and cognitive performance in T2DM patients with and without DKD adjusting for age, gender, and DDM.

	Non-DKD		DKD	
	R	*P*	R	*P*
MoCA	−0.227	0.005 ^*^	−0.099	0.361
DST	−0.074	0.370	−0.183	0.088
VFT	−0.169	0.040 ^*^	−0.204	0.056
CDT	−0.039	0.633	−0.080	0.460
TMTA	0.067	0.418	−0.107	0.321
TMTB	0.131	0.112	0.027	0.805
AVLT-IR	−0.171	0.037 ^*^	−0.106	0.325
AVLT-DR	−0.123	0.135	−0.057	0.601
LMT	−0.035	0.671	0.008	0.939

^*^P < 0.05. TyG, triglyceride glucose index; T2DM, type 2 diabetes mellitus; DKD, diabetic kidney disease; DDM, duration of diabetes mellitus; MoCA, Montreal cognitive assessment; DST, digit span test; VFT, verbal fluency test; CDT, clock drawing test; TMTA, trail making test-A; TMTB, trail making test-B; AVLT-IR, auditory verbal learning test-immediate recall; AVLT-DR, auditory verbal learning test-delayed recall; LMT, logical memory test.

### 3.5 Analysis for the risk factor for MCI in T2DM patients with and without DKD

To assess whether an increased TyG index independently contributes to the risk of MCI among individuals with T2DM, a binary logistic regression analysis was performed. The results demonstrated that, in participants without DKD, elevated TyG levels were significantly associated with a higher likelihood of MCI, even after adjusting for potential confounders such as age, sex, and DMM (OR: 2.236 and 2.245; both *p* = 0.004). However, in those with DKD, no independent association between TyG and MCI was observed, irrespective of covariate adjustment (all *p* > 0.05) (see Supplementary Table 2; Table 4).

**Table 4:** Assessment of risk factors for MCI by binary logistic analysis in T2DM patients with and without DKD adjusting for age, and DDM.

	Risk factors	*β*	*P*	OR	95% CI	
					Lower	Upper
Non-DKD	TyG	0.809	0.004 ^*^	2.245	1.298	3.884
	Gender	0.561	0.145	1.752	0.824	3.723
	Age	−0.009	0.697	0.991	0.946	1.038
	DMM	0.016	0.574	1.016	0.962	1.072
DKD	TyG	0.311	0.335	1.365	0.726	2.567
	Gender	0.189	0.689	1.208	0.480	3.041
	Age	0.021	0.397	1.022	0.972	1.073
	DMM	−0.033	0.302	0.968	0.910	1.030

^*^P < 0.05. MCI, mild cognitive impairment; T2DM, type 2 diabetes mellitus; DKD, diabetic kidney disease; DDM, duration of diabetes mellitus.

## 4 Discussion

In light of the rising prevalence of diabetes (5), this study undertook a comprehensive evaluation of MCI among individuals with T2DM, with a particular focus on metabolic determinants. The primary aim was to elucidate the association between the TyG index—a surrogate marker of metabolic dysfunction—and various cognitive domains, and to investigate whether this relationship differs in patients with and without DKD, an aspect not previously examined. Despite the cross-sectional design and lack of strict baseline matching between the MCI and non-MCI groups, individuals with cognitive impairment exhibited significantly higher levels of HbA1c and FPG, suggesting that suboptimal glycemic regulation may contribute to cognitive decline. This finding is consistent with earlier studies implicating hyperglycemia in the pathogenesis of diabetes-related cognitive deficits (12, 39, 40).

Although no marked differences were observed in lipid profiles between the two groups in our cohort, prior literature has reported associations between lipid parameters—such as LDL-c (41), HDL-c (42), and triglycerides (43, 44)—and cognitive outcomes. The discrepancy between our findings and earlier reports may stem from several factors: heterogeneity in study populations, non-linear relationships between lipid levels and cognition (e.g., U-shaped associations involving LDL-C) (45), and the potential necessity of composite biomarkers to capture the complexity of metabolic contributions to diabetic complications. For instance, ratios such as uric acid-to-HDL-c have previously been linked to diabetes-related outcomes (46). In this context, we further investigated the TyG index as a composite metabolic indicator. Our results revealed a significant elevation of the TyG index in the MCI group, suggesting a potential role of this index in cognitive dysfunction among T2DM patients. Given that the TyG index integrates fasting glucose and triglyceride concentrations, it is considered a proxy for insulin resistance and has gained prominence in assessing metabolic syndrome risk. Notably, higher TyG values were inversely correlated with cognitive performance, particularly in domains such as global cognition (measured by MoCA), executive function (via VFT), and short-term memory (assessed using AVLT-IR). These associations remained statistically robust even after adjusting for potential confounders including age, sex, and diabetes duration, supporting the utility of the TyG index as a candidate predictor of cognitive decline in T2DM.

Further stratified analyses revealed that the association between the TyG index and cognitive performance was more pronounced in individuals without DKD, whereas this relationship was not statistically significant among those with coexisting DKD. These findings suggest that renal function may play a pivotal role in the pathophysiology of diabetes-related cognitive impairment. On one hand, DKD itself may induce neurotoxic effects through pathways involving chronic inflammation (47, 48) and oxidative stress (28), potentially masking or overshadowing the contribution of metabolic burden—reflected by TyG—to cognitive decline. On the other hand, the presence of DKD may complicate metabolic signatures, thus diminishing the diagnostic accuracy of the TyG index within this subgroup. Complementary logistic regression analyses further demonstrated that, among T2DM patients without renal complications, elevated TyG levels were independently linked to MCI, even after adjusting for confounding variables such as age, sex, and disease duration. In contrast, this independent association was not observed in the DKD subgroup. These results underscore the need to consider renal status when investigating metabolic contributors to cognitive dysfunction in diabetes. Moreover, detailed cognitive assessments indicated that, in patients without DKD, higher TyG values were associated not only with impaired executive function but also with reduced immediate memory performance.

In summary, this study represents the first comprehensive examination of the association between the TyG index and cognitive impairment in individuals with T2DM. The findings suggest that this relationship is particularly pronounced among patients without DKD, indicating the potential utility of the TyG index as an early metabolic indicator for identifying individuals at elevated risk of cognitive decline in the diabetic population. Nevertheless, several limitations should be acknowledged. Firstly, as a cross-sectional study, our research did not apply strict matching for age and sex as would be done in a case–control design. Regarding this issue, we would like to clarify two points. (a) Our results showed no statistically significant differences in age or sex distribution between the two groups of participants. (b) Although such differences were not statistically significant, we nevertheless adjusted for age and sex in our subsequent analyses. While we have made every effort to minimize the potential bias arising from the lack of strict matching, we fully acknowledge that this remains one of the limitations of our study. Additionally, unlike a cohort study, it could not establish a causal relationship between the TyG index and MCI. These limitations are inherent to the nature of our study. Secondly, our study did not report the potential effects of medications. In fact, during the study design stage, we had already considered the influence of drug use and collected relevant medication information from participants. These included antidiabetic agents (e.g., metformin, insulin, SGLT2 inhibitors, and GLP-1 receptor agonists), lipid-lowering drugs (such as statins), and antiplatelet agents (such as aspirin). However, unfortunately, we only recorded whether participants were using a particular class of drugs, without obtaining detailed information on dosage, duration, or treatment adherence. As our study was based on a relatively small sample size, and some medications were used by only a few participants. Therefore, it was difficult to assess the specific impact of drug use with the available data, which represents a limitation of our study. Interestingly, a recent network meta-analysis comprehensively evaluated the effects of antidiabetic agents on cognitive function, highlighting the potential neuroprotective roles of SGLT2 inhibitors and GLP-1 receptor agonists (49). Thirdly, regarding smoking and alcohol consumption, because both are strongly associated with diabetic complications, patients with such histories were excluded at the time of enrollment. We acknowledge, however, that exposure to secondhand smoke is also relevant to diabetic complications. Unfortunately, data on secondhand smoke exposure were not collected in our study, which we recognize as a limitation. Fourth, the absence of key biological markers—such as pro-inflammatory mediators, oxidative stress indicators, and neurobiological factors—restricts the mechanistic insight that could be drawn from the results. Future investigations should employ longitudinal approaches to further evaluate the predictive capacity of the TyG index, ideally integrating neuroimaging techniques, biomolecular profiling, and immunological markers to establish a more robust and multidimensional risk stratification framework for diabetes-related cognitive dysfunction.

## 5 Conclusion

In conclusion, this study demonstrates that higher levels of the TyG index are independently associated with an elevated risk of developing MCI in individuals diagnosed with T2DM who do not exhibit DKD. Importantly, the strength and nature of the association between TyG and cognitive decline appear to differ depending on the presence of DKD. Among T2DM patients without DKD, TyG may serve as valuable early markers for identifying those at risk for MCI. Moreover, elevated TyG in this subgroup was linked to deficits in executive function as well as impairments in short-term memory performance. Although we did not directly assess TyG as a biomarker for MCI in T2DM patients here, we consider it one of the priority directions for future research. Accordingly, we believe that TyG may serve as a potential biomarker for MCI in T2DM patients in future clinical applications.

## Data Availability

The original contributions presented in the study are included in the article/Supplementary material, further inquiries can be directed to the corresponding authors.
