# Supplementary material for: Different association between triglyceride-glucose index and mild cognitive impairment in type 2 diabetes mellitus patients with and without diabetic kidney disease
Source: Front Nutr. 2025 Nov 12;12:1681164. doi: 10.3389/fnut.2025.1681164 (PMC12649707; doi:10.3389/fnut.2025.1681164)
Supplement: Supplementary file 1 [file Table_1.docx]

Supplementary-Table1: Pearson association between TyG and cognitive performance in T2DM patients with and without DKD

| TyG | Non-DKD | | DKD | |
| --- | --- | --- | --- | --- |
|  | R | P | R | P |
| MoCA | -0.216 | 0.008 ^*^ | -0.077 | 0.470 |
| DST | -0.073 | 0.369 | -0.196 | 0.062 |
| VFT | -0.180 | 0.026 ^*^ | -0.200 | 0.057 |
| CDT | -0.032 | 0.696 | 0.004 | 0.971 |
| TMTA | 0.053 | 0.513 | 0.089 | 0.403 |
| TMTB | 0.129 | 0.113 | 0.034 | 0.746 |
| AVLT-IR | -0.169 | 0.037 ^*^ | -0.093 | 0.378 |
| AVLT-DR | -0.127 | 0.118 | -0.044 | 0.679 |
| LMT | -0.023 | 0.776 | 0.092 | 0.388 |

Notes: ^*^ P<0.05. Abbreviations: TyG, triglyceride glucose index; T2DM, type 2 diabetes mellitus; DKD, diabetic kidney disease; MoCA, Montreal cognitive assessment; DST, digit span test; VFT, verbal fluency test; CDT, clock drawing test; TMTA, trail making test-A; TMTB, trail making test-B; AVLT-IR, auditory verbal learning test-immediate recall; AVLT-DR, auditory verbal learning test-delayed recall; LMT, logical memory test.
