# Supplementary material for: Different association between triglyceride-glucose index and mild cognitive impairment in type 2 diabetes mellitus patients with and without diabetic kidney disease
Source: Front Nutr. 2025 Nov 12;12:1681164. doi: 10.3389/fnut.2025.1681164 (PMC12649707; doi:10.3389/fnut.2025.1681164)
Supplement: Supplementary file 2 [file Table_2.docx]

Supplementary -Table 2: Assessment of risk factors for MCI by binary logistic analysis in T2DM patients with and without DKD

|  | β | P | OR | 95% CI | |
| --- | --- | --- | --- | --- | --- |
|  |  |  |  | Lower | Upper |
| Non-DKD | 0.805 | 0.004 ^*^ | 2.236 | 1.301 | 3.844 |
| DKD | 0.312 | 0.301 | 1.367 | 0.756 | 2.471 |

Notes: ^*^ P<0.05. Abbreviations: MCI, mild cognitive impairment; T2DM, type 2 diabetes mellitus; DKD, diabetic kidney disease.
